# Identification and analysis of the AP2/ERF gene family in *Dendrobium officinale* based on pan-genome and functional characterization of *DofERF109_2*

**DOI:** 10.3389/fpls.2026.1834268

**Published:** 2026-06-10

**Authors:** Zhibo Zhang, Zhenyu Hou, Shuying Zhu, Siyun Wei, Songping Li, Qingyun Xue, Wei Liu, Xiaoyu Ding, Zhitao Niu

**Affiliations:** 1 College of Life Sciences, Key Laboratory of State Forestry and Grassland Administration for Dendrobium officinale, Nanjing Normal University, Nanjing, China; 2 Jiangxi Institute of Red Soil and Germplasm Resources, Nanchang, Jiangxi, China; 3 School of Life and Health Sciences, Huzhou College, Huzhou, Zhejiang, China; 4 Shenzhen Key Laboratory for Orchid Conservation and Utilization, The National Orchid Conservation Center of China and The Orchid Conservation and Research Center of Shenzhen, Shenzhen, China

**Keywords:** anthocyanidin, AP2/ERF, *Dendrobium officinale*, *DofERF109_2*, pangenome family

## Abstract

**Introduction:**

AP2/ERF transcription factors are key regulators of plant stress responses and developmental processes. Despite their functional significance, limited research has focused on this gene family in the medicinal orchid *Dendrobium officinale*.

**Methods:**

Based on the pangenome data of seven *Dendrobium officinale* individuals from different habitats, we performed a pangenome family analysis of AP2/ERF, including analyses of presence-absence variation (PAV), selection pressure, transposable elements, etc., and conducted functional validation of the screened key members.

**Results:**

A total of 101, 76, 113, 123, 113, 105 and 113 AP2/ERF genes were identified in the seven *Dendrobium officinale* individuals, respectively. PAV analysis classified the non redundant members into core (29), softcore (28), dispensable (17) and private (3) genes. Compared with *Arabidopsis thaliana*, *D. officinale* AP2/ERFs exhibited a significant evolutionary contraction, although some genes underwent duplication. Most genes experienced negative selection, while a few showed positive selection. Cold and heat stress induced differential expression patterns; genes with stable expression were predominantly core or softcore members. The candidate *DofERF109_2* localized to the nucleus. Its transient expression suppressed anthocyanin accumulation in tobacco and downregulated the key enzyme gene *DofCHI* in *D. officinale*.

**Discussion:**

Our pan genome analysis demonstrates that the AP2/ERF family in *D. officinale* has undergone significant evolutionary contraction compared with Arabidopsis, suggesting lineage specific gene loss or rapid divergence in orchids. Despite this overall contraction, lineage specific duplications (e.g., *ERF109* and *RAP2.11*) were observed, which may provide raw material for adaptive evolution. The classification into core, softcore, dispensable and private genes reveals a conserved set likely involved in essential functions, whereas variable genes may contribute to local adaptation. Ka/Ks analysis identified positive selection only in a few genes, often those with recent duplications, supporting neofunctionalization. The widespread presence of transposable elements (68.8% of members) suggests that TE insertion is a common, ongoing process that may generate regulatory diversity without being strongly counter selected. Expression profiling under temperature stress further highlighted functional divergence: cold stress induced gradual upregulation, while heat stress caused downregulation of most genes. Notably, the nuclear localized *DofERF109_2* negatively regulated anthocyanin biosynthesis by suppressing *DofCHI* expression and reducing pigment accumulation. Together, our results provide a comprehensive pan genome resource of AP2/ERFs in *D. officinale* and identify *DofERF109_2* as a candidate negative regulator of anthocyanin synthesis.

## 1 Introduction

*Dendrobium* plants are widely distributed throughout tropical and subtropical Asia as well as Oceania (Tang et al., 2017; Zheng et al., 2025). *Dendrobium* plants typically grow attached to other plants or rock surfaces and are adapted to shaded environments (Yang et al., 2025), a characteristic that endows them with various medicinal bioactive constituents, including polysaccharides, dendrobine, and flavonoids. *Dendrobium officinale* is renowned as the “foremost of the nine immortal herbs” (Zhang et al., 2023), processed using traditional methods, it can be made into a health-promoting product., and studies have confirmed that extracts of *D. officinale* have a mitigating effect on gastric injury in rats induced by external drugs (Wang et al., 2025). The availability of a chromosome-level reference genome for *D. officinale*, referred to as “Niu2020”, represents a landmark resource for genomic studies in this species (Niu et al., 2021; Zheng et al., 2025). It has laid the foundation for functional gene mining and comparative genomics analyses in *D. officinale*. Recent research has constructed a GFP-mediated CRISPR-Cas9 system for *D. officinale* (Li et al., 2025b), providing instrumental support for further investigation into how particular genes contribute to the physiological traits of *D. officinale*. The current standards for identifying the quality of *D. officinale* (Sun et al., 2024) are based on the types and content levels of polysaccharides, monosaccharides, and flavonoids. Therefore, in addition to polysaccharides, flavonoids are also crucial for *D. officinale* to exert specific pharmacological effects, and the involvement of flavonoids in enhancing plant stress resistance is well documented (He et al., 2023).

Flavonoids can be divided into seven classes of compounds (Shen et al., 2022). Among these, anthocyanidins possess multiple biological functions in plants (Zhang et al., 2026). During the reproduction of entomophilous plants, anthocyanidins can serve as plant pigments, assisting flowers and fruits in attracting insects, thereby facilitating pollination and seed dispersal. Furthermore, studies have shown that anthocyanins can scavenge reactive oxygen species generated by ultraviolet radiation in plants (Shi et al., 2023), reducing their damage to plant organs. During biosynthesis in plants, the anthocyanin synthesis pathway is conserved (Davies et al., 2024; He et al., 2026) and is regulated by various rate-limiting enzymes.

Transcription factors belonging to the AP2/ERF family play indispensable roles in plant biology (Magnani et al., 2004; Ma et al., 2024; Zhu et al., 2024). Their classification hinges upon a characteristic AP2/ERF structural domain, which is responsible for facilitating protein-DNA interactions. Emerging evidence suggests an evolutionary link between plant AP2/ERF transcription factors and HNH-AP2 endonucleases of ancient prokaryotic or viral origin (Zhu et al., 2024). The classification of the AP2/ERF superfamily (Sakuma et al., 2002; Nakano et al., 2006) relies on conserved domain composition. This scheme delineates four distinct families: AP2, ERF, RAV, and Soloist. Proteins in the AP2 family are defined by tandem AP2/ERF domains. The ERF family, characterized by a single ERF domain, is itself partitioned into the ERF and DREB subfamilies according to sequence divergence, a distinction that confers differential binding to specific cis-acting elements. RAV family members uniquely combine an AP2/ERF domain with a B3 domain. The Soloist family comprises proteins with an atypical AP2/ERF-like domain whose structure differs markedly from those of the other groups. Advances in high-throughput sequencing have enabled comprehensive, genome-wide analyses of the AP2/ERF gene family in numerous plants. These species include the model organism *Arabidopsis thaliana* (Nakano et al., 2006), as well as important crops like rice (Nakano et al., 2006), grape (Zhu et al., 2019), and maize (Hao et al., 2020).

Numerous investigations have established the AP2/ERF family as a key player in developmental processes, plant growth, and responses to environmental stimuli (Feng et al., 2020; Tang et al., 2024). Its fundamental importance in enabling plants to withstand external pressures is supported by multiple lines of evidence (Qu et al., 2020; Xie et al., 2022; Wei et al., 2025). While the MBW transcriptional complex(a trimer composed of members of the MYB, bHLH, and WD40 families) occupies a central role in the regulatory network of plant anthocyanin biosynthesis (Qi et al., 2020), emerging evidence indicates that members of the AP2/ERF also participate in modulating this pathway through diverse molecular mechanisms. Regarding direct binding to key enzyme genes in the anthocyanin pathway, experimental evidence in blueberry has demonstrated that the *VcCRF9* protein directly interacts with *VcANS* (Ma et al., 2026), while direct interaction between *NtERF13a* and the *NtF3’H* and *NtANS* proteins has been observed in tobacco (Wang et al., 2023). Concurrently, interactions between AP2/ERF and MYB transcription factors have also been documented in plants, indirectly regulating anthocyanin synthesis in plants such as *Pyrus* (Ni et al., 2019), Sichuan pepper, and eggplant (Li et al., 2025a). Furthermore, as components of hormone signaling pathways, AP2/ERF members can respond to various hormone signals, including melatonin (Sun et al., 2025), ABA (Ma et al., 2026), and ethylene (Zhang et al., 2018), indicating that AP2/ERF members can serve as molecular bridges between hormone signaling and anthocyanin accumulation.

In summary, AP2/ERF proteins are implicated in a broad spectrum of plant biological functions, particularly in encompassing developmental regulation, defense against environmental stresses, and anthocyanin biosynthesis; however, related research in *D. officinale* is scarce, and there is an urgent need to conduct such studies. The AP2/ERF gene family was characterized in the present work through comparative analysis of seven *D. officinale* genomes and performed analyses of selection pressure, transposable elements, and expression profiles under cold and heat stress; we screened out *DofERF109_2* from *D. officinale* and conducted functional validation, including subcellular localization and transient expression experiments. These results fill a gap in pangenome family research on *D. officinale* and also provide candidate genes for dissecting the regulatory architecture underlying anthocyanin accumulation in *D. officinale*; future research could conduct further protein interaction experiments on the screened gene to investigate how this factor specifically governs anthocyanin accumulation at the molecular level.

## 2 Materials and methods

### 2.1 Plant material

The materials utilized in this research were sourced from the Institute of Plant Resources and Environment, Nanjing Normal University. We selected seven *D. officinale* individuals from different habitats; their designations, collection locations, and geographic coordinates are as follows: (a) Dof (Huoshan, Anhui) (31.38°N, 116.32°E); (b) HXL (Huoshan, Anhui) (31.00°N, 115.00°E); (c) TM (Tianmushan, Zhejiang) (30.18°N, 119.23°E); (d) TP1 (Leiqing, Zhejiang) (28.07°N, 120.57°E); (e) TP4 (Danxiashan, Guangdong) (28.07°N, 113.36°E); (f) hs (Huoshan, Anhui) (30.43°N, 116.27°E); (g) YD (Yandangshan, Zhejiang) (30.43°N, 116.27°E).

### 2.2 Genomic DNA isolation and high-throughput sequencing

Fresh young leaves of *D. officinale* were selected, and a modified CTAB method (Xu et al., 2012) was employed to extract high-quality genomic DNA. Samples with high purity were selected to construct short DNA fragment libraries.Then we employed next-generation (Illumina HiSeq 2500) and single-molecule real-time (PacBio Sequel II) sequencing technologies. Quality control of the generated sequences was carried out according to the standard pipeline implemented in SMRT Link version 8.0 to obtain high-quality DNA sequence data. **Supplementary Table 1** lists all gene sequences examined in this work; the corresponding genome files were derived from our team’s earlier publication (Niu et al., 2021).

### 2.3 Genome assembly and annotation

With the assistance of Hi-C data, the sequenced reads were assembled using Hifiasm (v0.18.5) through all-versus-all alignment and multiple rounds of error correction to construct a complete genome assembly. During this process, The quality of the final genome assembly was evaluated with BUSCO (Simão et al., 2015) to confirm its completeness and precision.

### 2.4 Analysis of AP2/ERF family genes: identification and property evaluation

We employed a combined approach using HMM (Potter et al., 2018) and BLASTp (Camacho et al., 2009) for genome-wide detection of AP2/ERF genes. HMMER searches were operated against the *D. officinale* genomes using the conserved domain models for AP2/ERF (PF00847) and B3 (PF02362) (Mistry et al., 2021) for identification and screening. Concurrently, we obtained the entire set of AP2/ERF transcription factor genes in *A. thaliana* and performed BLASTp searches against the genomes of different *D. officinale* individuals; we then took the union of the genes identified by both the HMM and BLASTp screens. Subsequently, conserved motifs and functional domains of the candidate genes were characterized using the NCBI (Lu et al., 2020) as well as the SMART website web server (Letunic et al., 2021). We used Protparam (Wilkins et al., 1999) for performing physicochemical property analysis.

### 2.5 Phylogenetic analysis of AP2/ERF

Using ClustalX (Larkin et al., 2007), we performed sequence alignments of AP2/ERF candidates. The aligned sequences were then used to construct a NJ tree with MEGA X (Kumar et al., 2018) Following the phylogenetic framework established for *A. thaliana*, we systematically classified the pangenome-wide AP2/ERF genes identified in *D. officinale* (Nakano et al., 2006).

### 2.6 Presence-absence variation of AP2/ERF members in seven *D. officinale* individuals

Based on the identification results described above, the distribution patterns of different gene members across the seven individuals were integrated and compiled into a matrix; subsequently, a heatmap was constructed.

### 2.7 Ka/Ks and chromosomal localization analysis

We paired individuals that were classified as homologous to the same Arabidopsis thaliana member into gene pair files, then used the Simple Ks/Ks Calculator (NG) in TBtools (Chen et al., 2023) to perform Ka/Ks analysis, and organized the resulting data into a matrix for heatmap construction. Based on the AP2/ERF family identification results and the GFF files of different family members, we performed chromosomal localization mapping of the genes using the Gene Location Visualize from GTF/GFF tool in TBtools.

### 2.8 Transposable Element Analysis

We used EDTA (Extensive *de-novo* TE Annotator) to identify transposable elements in the genome. Data were preprocessed, and transposable element annotation was performed using default parameters, generating two output files: a summary file and a GFF file. The former displays the types and quantities of transposable elements, while the latter records the specific locations of transposable elements within the genome. Based on the transposable element identification results and the AP2/ERF family identification results, we compiled the numbers of different members containing transposable elements, formed a matrix, and generated a plot.

### 2.9 Subcellular localization prediction

The amino acid sequences of all *D. officinale* candidate members were submitted to the CELLO v.2.5 webserver (Yu et al., 2004) for subcellular localization analysis; the subcellular localization result with the highest algorithm score was selected.

### 2.10 Transcriptome data acquisition and analysis

TRIzol-based extraction yielded total RNA from *D. officinale*. RNA quality was assessed using an Agilent 2100 Bioanalyzer. The transcriptome data were derived from temperature stress experiments on *D. officinale* conducted by our research group, with cold stress at 4 °C, standard condition at 22 °C, and heat stress at 30 °C. DESeq2 was employed for differentially expressed gene (DEG) with p-adj < 0.05 and |log2FC| ≥ 1 analysis (Love et al., 2014).

### 2.11 Construction of the expression pattern of *DofERF109_2* under temperature stress

*D. officinale* plants were subjected to different temperature conditions (cold stress at 4 °C and heat stress at 30 °C). Leaves were collected at various time points after treatment (4 h, 8 h, 12 h, 16 h, 20 h, 24 h), immediately frozen in liquid nitrogen, and subjected to qRT-PCR to quantify the expression level of *DofERF109_2*. Four biological replicates and three technical replicates were performed for each experimental group. The results were analyzed using one-way ANOVA. (The primers for the reference gene were: F: TTCGGAAGGATTGGAAGGCTTGTAG; R: GAGATGATAACCTTCTTGGCACCGC).

### 2.12 Subcellular localization assay

Primers flanking *DofERF109_2* (F: ATGGCGTTCAATCAGCAACTCAA; R: CAATCCGCCCATCTCCTCTCC) were designed for gene amplification. The amplified product was ligated into the pCambia1300-35S-EGFP empty vector. The two constructs were subsequently introduced into GV3101(*Agrobacterium tumefaciens*). After activation of the bacterial colonies, the transformation solution was adjusted to an OD_600_ of 0.8, supplemented with acetosyringone (AS), and allowed to stand in the dark for 3 hours. Subsequently, the solution was injected into *Nicotiana benthamiana* leaves. Confocal laser scanning microscopy was employed to visualize fluorescence signals.

### 2.13 Transient expression experiment of *DofERF109_2* in the *D. officinale*

The target gene was amplified using the primers described above, ligated into the pCAMBIA1301-35SN plasmid via homologous recombination, and transformed into Agrobacterium tumefaciens strain GV3101. After activation of the bacterial colonies, the transformation solution was adjusted to an OD_600_ of 0.8, supplemented with acetosyringone (AS), and allowed to stand in the dark for 3 hours. The transformed Agrobacterium was then injected into *D. officinale* leaves, followed by a 12-hour dark treatment. Leaf tissues were collected on the third day after injection for qPCR analysis. For each group, four biological replicates (four leaves) and three technical replicates (three experiments using total RNA from the same leaf) were performed. The relative expression levels between different gene treatment groups and the control group were statistically analyzed using two-way ANOVA.(The primers for the reference gene were: F: TTCGGAAGGATTGGAAGGCTTGTAG; R: GAGATGATAACCTTCTTGGCACCGC).

### 2.14 Transient expression experiment of *DofERF109_2* in the tobacco

*DofERF109_2*, *DofMYB47*, *Rosea1*(GenBank: DQ275529.1), and *AtANS* were homologously recombined into the pCAMBIA1301-35SN plasmid and transformed into Agrobacterium tumefaciens strain GV3101. After activation of the bacterial colonies, the transformation solution was adjusted to an OD_600_ of 0.8, supplemented with acetosyringone (AS), and allowed to stand in the dark for 3 hours. Two combinations at the indicated ratios (positive control:*Rosea1*:*AtANS* = 1:1;negative control:pCAMBIA1301-35SN empty vector;experimental group:*DofERF109*:*DofMYB47*:*AtANS* = 1:1:1; *DofMYB47*:*AtANS* = 1:1) were used to infiltrate tobacco leaves. The positive control was the *Roseal: AtANS* = 1:1 combination, and the negative control was infiltrated with the empty pCAMBIA1301-35SN plasmid alone. Photographs were taken 7 days after infiltration to observe the results.2.15 Extraction of anthocyanins from tobacco leaves.

Prepare anthocyanin extraction solution (for example, 100 mL: 18 mL 1-propanol + 3 mL 15% HCl + 79 mL ddH_2_O). Cut the corresponding tobacco leaf area, weigh (fresh weight), add 1 mL of the above extraction solution, incubate in a boiling water bath for 3 min, then keep at room temperature (25 °C) in the dark overnight (12 h), and centrifuge at 18,000 rpm (39,000 × g) for 20 min. Measure A_535_ and A_650_ using a spectrophotometer. Anthocyanin content = (A_535_ - A_650_)/fresh weight of leaf (g). Statistical analysis of anthocyanin content among different treatment groups was performed using one-way ANOVA.

## 3 Results

### 3.1 Identification of AP2/ERFs in the *D. officinale* pangenome

**Figure 1:**
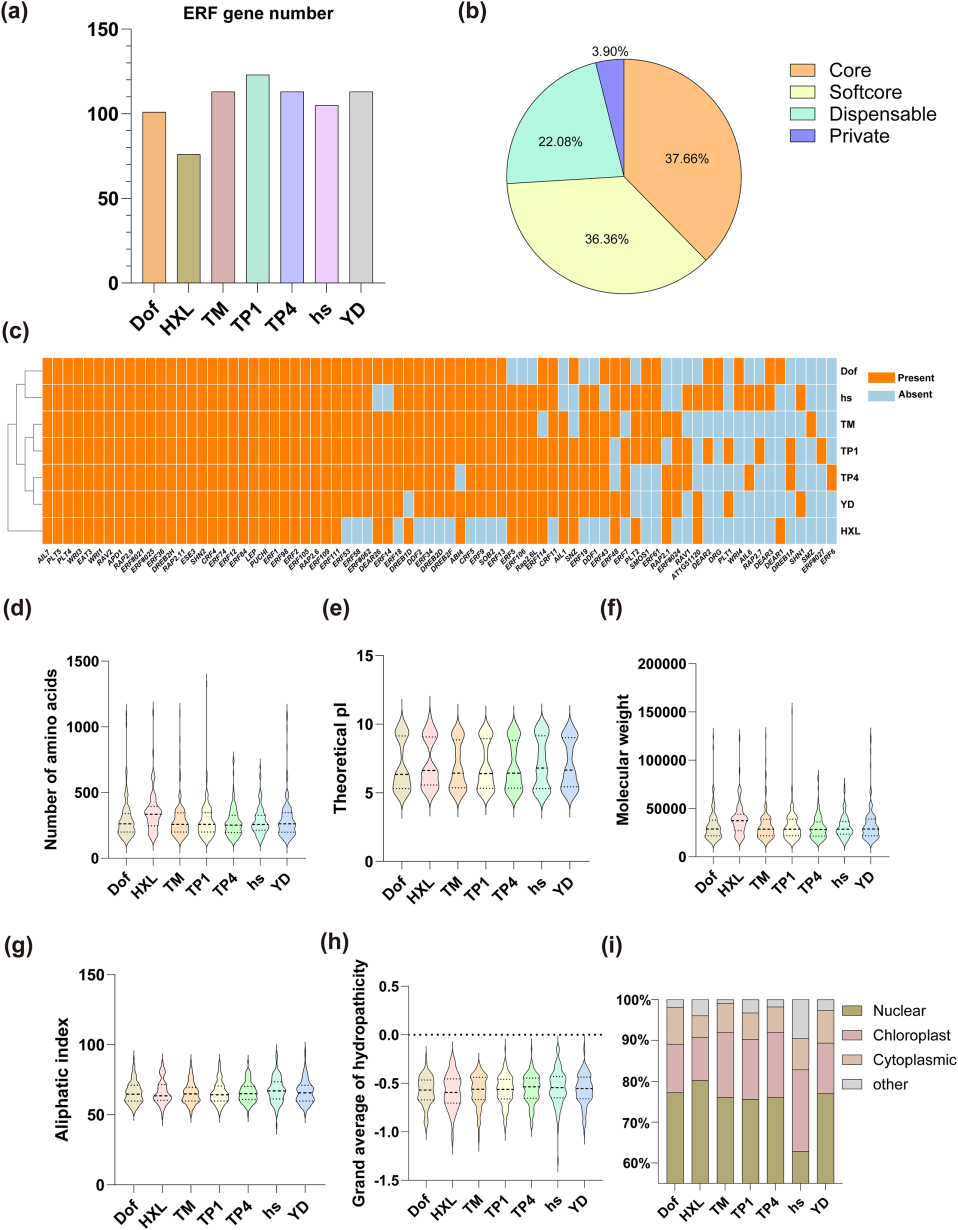
Pangenome-wide discovery and physicochemical assessment of AP2/ERFs in *D. officinale*: **(A)** Number of AP2/ERFs in different individuals. **(B)**Distribution of core, softcore, dispensable, and private genes. **(C)** Presence-absence variation results of AP2/ERF members (orange indicates present, blue indicates absent). **(D)** Number of amino acids of AP2/ERFs in different individuals. **(E)** Theoretical pI of AP2/ERF member proteins in different individuals. **(F)** Molecular weight of AP2/ERF member proteins in different individuals. **(G)** Aliphatic index of AP2/ERF member proteins in different individuals. **(H)** Grand average of hydropathicity of AP2/ERFs in different individuals. **(I)** Subcellular localization prediction results of AP2/ERF members in different individuals.

With reference to the AP2/ERF family genes of *A. thaliana*, combined with HMMER, BLASTp methods, a complete set of AP2/ERF genes was detected in every one of the seven *D. officinale* specimens analyzed, screening out 101, 76, 113, 123, 113, 105, and 113 genes, respectively (**Figure 1a**). The gene family showed variation in size across individuals, with the highest number observed in TP1 and the lowest in HXL. We categorized the identified genes according to their distribution patterns: core genes were present in every individual; softcore genes appeared in five or six; dispensable genes were found in two to four; and private genes occurred in only one individual. Through homology identification and statistical analysis, the seven *D. officinale* samples collectively harbored 77 members of the AP2/ERF gene family (**Figure 1b**); these comprised 29 core genes (accounting for 37.66%), 28 softcore genes (36.36%), 17 dispensable genes (22.07%), and 3 private genes (3.90%).

We found that many ERF family members exhibited duplication events in *D. officinale*, as shown in **Supplementary Table 2**; similarly, some ERF members from *A. thaliana* did not have homologous genes identified in *D. officinale*. The distribution of each ERF family member across the seven *D. officinale* individuals is illustrated in **Figure 1c**, and **Supplementary Table 2** provides a detailed breakdown of the numbers corresponding to each ERF family member detected in the *D. officinale* pangenome; the individuals containing private genes were TM, TP1, and TP4. To further characterize the AP2/ERF candidates, we assessed their physicochemical features, which are illustrated in **Figures 1d-h**, with the resulting data provided in **Supplementary Table 3**.

### 3.2 Evolutionary relationships of AP2/ERF genes in the *D. officinale* pangenome

**Figure 2:**
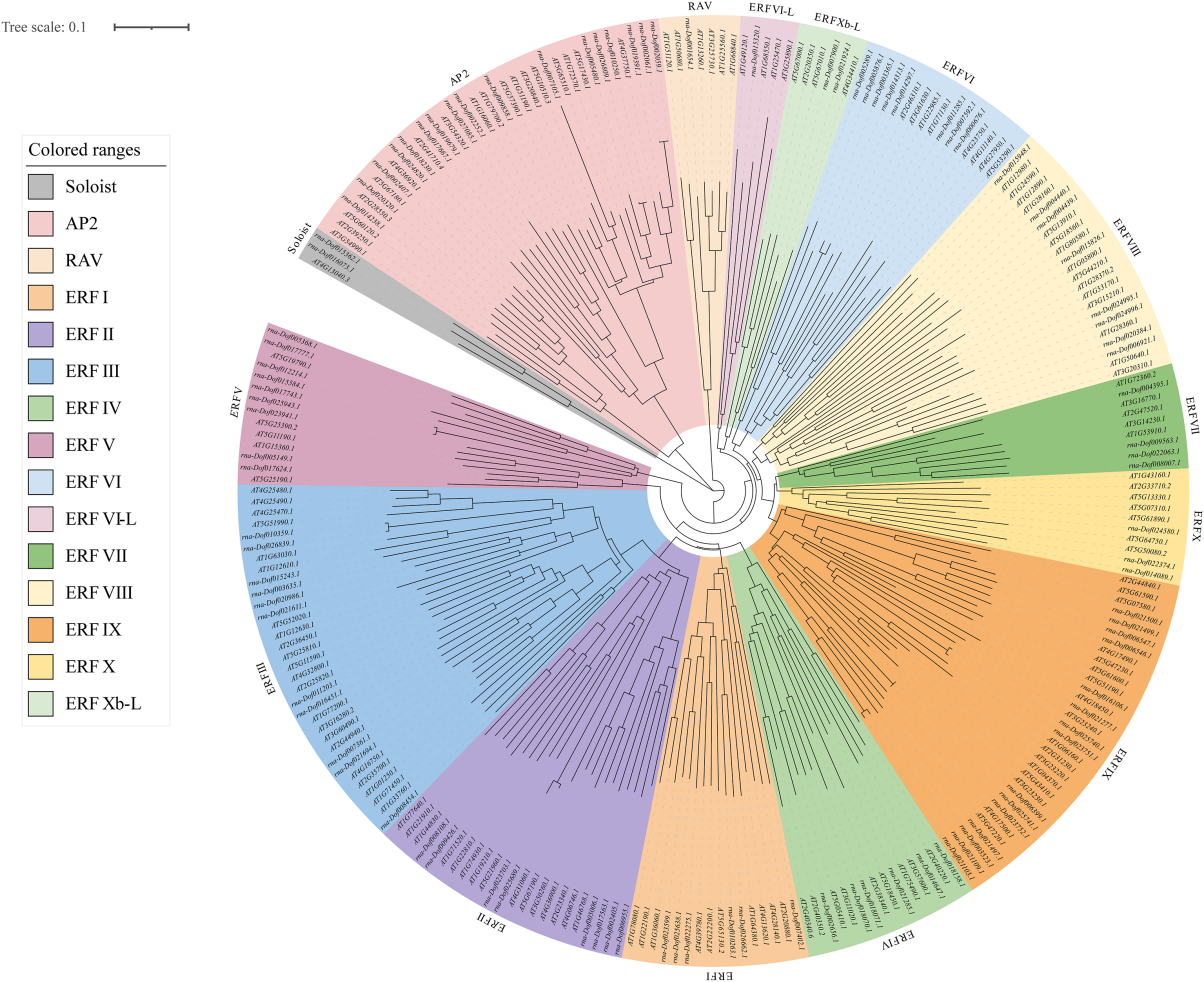
Evolutionary relationships of AP2/ERFs in Dof individuals and reference plant (using the Neighbor-Joining method after sequence alignment with ClustalX in MEGA).

**Figure 3:**
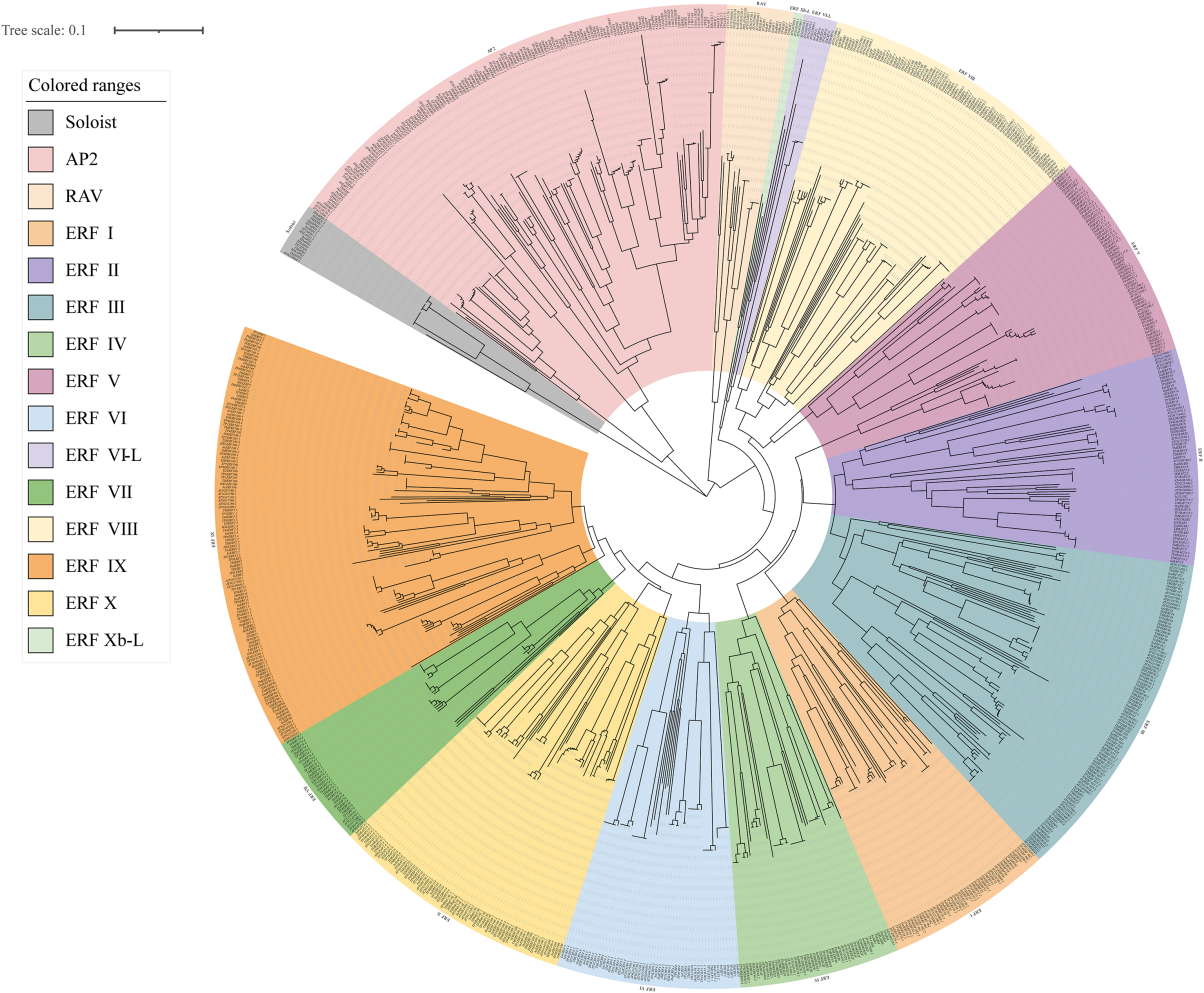
Evolutionary relationships of AP2/ERFs in *D. officinale* and reference plant (using the Neighbor-Joining method after sequence alignment with ClustalX in MEGA).

Using the amino acid sequences of AP2/ERF proteins from *D. officinale* and *A. thaliana*, we generated a phylogenetic tree. Based on the previously published classification of the AP2/ERF family, the members from *D. officinale* were categorized into the following 15 groups: Soloist, AP2, RAV, and ERF (I-X, VI-L, Xb-L). Among these, ERFI-ERFIV belong to the DREB subfamily, while ERFV-ERFX belong to the ERF subfamily. The phylogenetic tree is shown in **Figures 2**, **3**. We assigned gene names to the AP2/ERF family members within the *D. officinale* pangenome based on the phylogenetic tree and BLASTp results. With the exception of the ERF Xb-L subfamily, all other subfamilies contained homologous genes in *D. officinale*. Among the seven *D. officinale* individuals, members of the AP2 subfamily were the most numerically predominant among all identified groups, while the Soloist, ERFVI-L, and ERFVII subfamilies each contained one family member. Based on the pangenome and phylogenetic analysis results, core genes were present in all subfamilies except for ERFI, ERFVI-L, and ERF Xb-L.

### 3.3 Analysis of selective pressures acting on AP2/ERF family members across different *D. officinale* individuals

**Figure 4:**
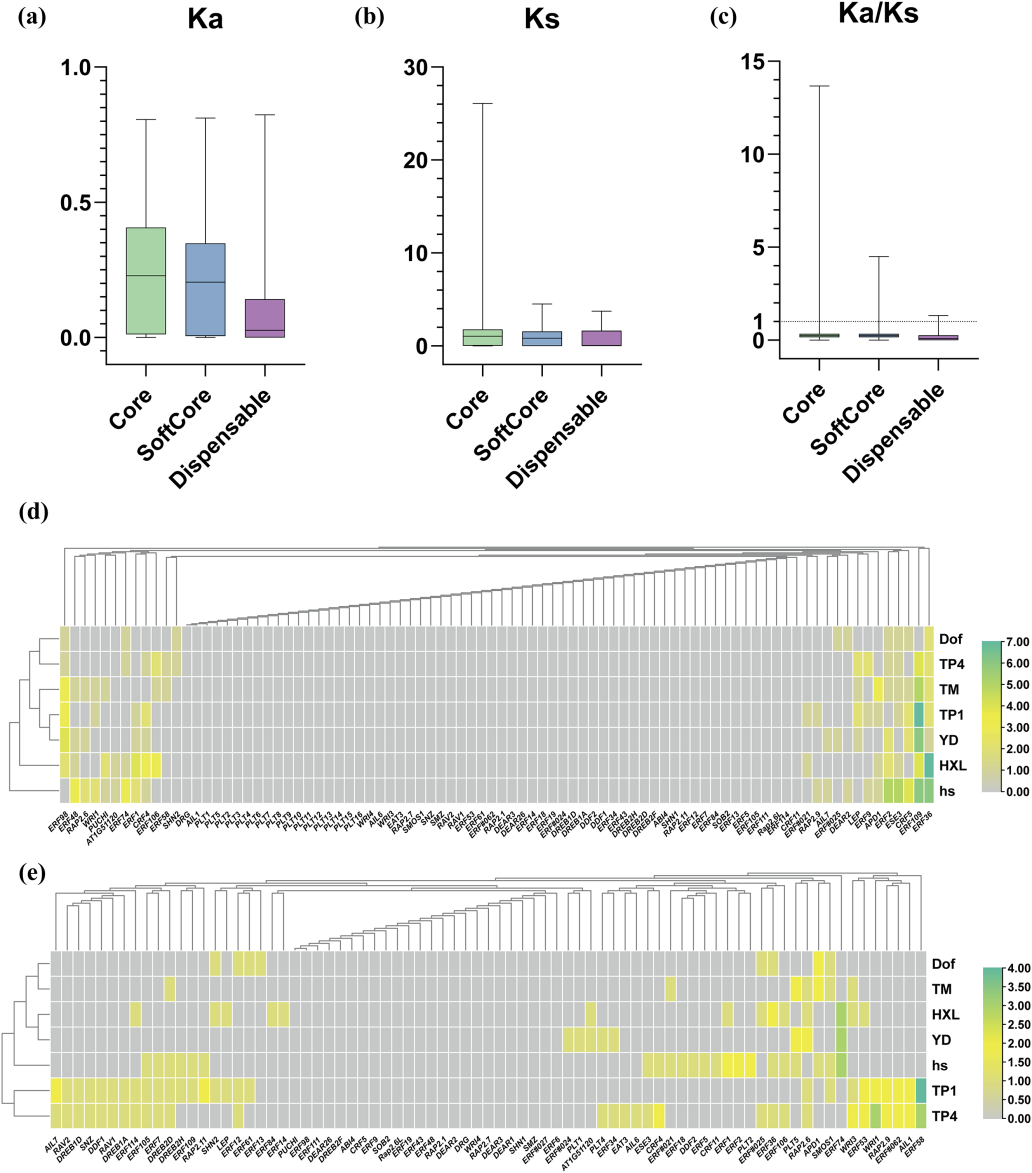
Selection pressure and transposable element analysis of AP2/ERF family members in *D. officinale*: Distribution of **(A)** Ka, **(B)** Ks, and **(C)** Ka/Ks ratios in core, softcore, and dispensable genes. **(D)** Distribution of genes with Ka/Ks > 1 (gray indicates genes with all Ka/Ks ratios < 1). **(E)** Distribution of transposable elements among AP2/ERF members (gray indicates members in which no transposable element was identified).

The Ka/Ks analysis results for all ERF gene members are presented in **Supplementary Table 4**. We compiled the distribution patterns of Ka, Ks, and their ratio (Ka/Ks) for core, softcore, and dispensable genes (Ka/Ks ratios could not be calculated for private genes) (**Figures 4a–c**). Among the core, softcore, and dispensable genes, we identified a total of 25 genes with Ka/Ks > 1 across the different individuals, distributed across 14 subfamilies. These genes likely provide a competitive advantage for the individuals in their specific habitats. The distribution of positively selected genes among the individuals is shown in **Figure 4d**, where grey indicates that the gene did not exhibit Ka/Ks > 1 in that particular individual.

### 3.4 Transposable element analysis of the AP2/ERF members in the *D. officinale* pangenome

To analyze the potential contribution of transposable elements to the evolution of ERF genes, we performed transposable element analysis on AP2/ERF family members from different *D. officinale* individuals, as shown in **Figure 4e**. A total of 53 genes contained transposable element fragments in different individuals; these genes were distributed across 14 AP2/ERF subfamilies, accounting for 68.8% of the overall count of AP2/ERF genes(77 members). Therefore, we speculate that transposable element insertion is a widespread and continuous event in the evolution of the gene members, and that natural selection has not forcibly eliminated these elements; they may be a result of neutral evolution. Except for private genes, members carrying fragments of transposable elements were identified in every one of the other three gene groups. Among the core genes, all except *DofPUCHI*, *DofERF98*, and *DofERF111* contained transposable element fragments.

### 3.5 Analysis of the chromosomal locations of AP2/ERF genes

**Figure 5:**
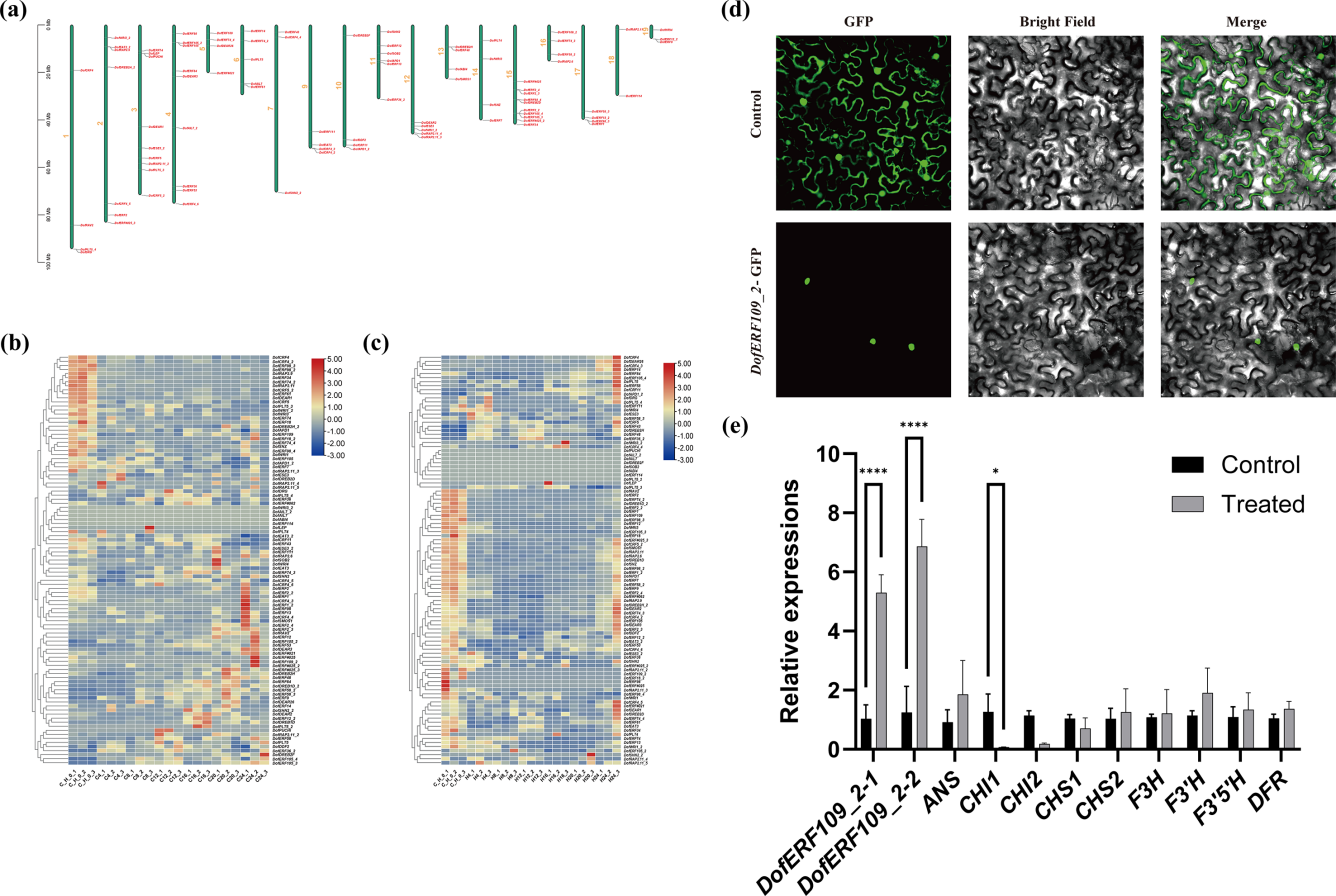
Chromosomal localization, expression profiles, and functional validation of *DofERF109_2* in AP2/ERF members.: **(A)** Chromosomal localization of AP2/ERF members in the Dof individual. **(B)** Transcriptional responses of *D. officinale* AP2/ERFs under cold stress. **(C)** Transcriptional responses of *D. officinale* AP2/ERFs under heat stress. **(D)** Subcellular localization results of *DofERF109_2*. **(E)** The results of transient expression experiments of *DofERF109_2*. *P ≤ 0.05, ****P ≤ 0.0001.

The chromosomal localization map for AP2/ERF family members in the Dof individual is shown in **Figure 5a**, while the chromosomal localization information for the remaining individuals is summarized in **Supplementary Figure 1**. We found that, except for 9, 2, and 4 genes from the Dof, TM, and TP1 individuals, respectively, which were not localized to the 19 chromosomes, all other genes were mapped to specific chromosomes. The AP2/ERF members not localized to chromosomes are summarized in **Supplementary Table 5**. In a few regions, we observed tandem duplication pairs composed of 2 to 4 genes; for example, on Chr15 of the Dof individual, there is one tandem duplication pair consisting of four genes.

Through comparative genomic analysis, we found that the chromosomal distribution of the ERF gene family is generally conserved. Notably, all AP2/ERF members in the Dof individual were localized to 18 of the 19 chromosomes, with no AP2/ERF family members found on chromosome 8; in contrast, AP2/ERF genes in the other six individuals were distributed across all 19 chromosomes. This absence of chromosomal localization might be attributed to factors such as assembly differences or sequencing depth.

### 3.6 Transcriptional profiling of AP2/ERF genes across the *D. officinale* pangenome

To gain insight into the functional relevance of AP2/ERF genes during temperature pressure, we profiled the transcriptional responses of all family members in the Dof individual under diverse thermal treatments, as summarized in **Figures 5b, c**. The treatment conditions included low temperature (4°C) for 4–24 h (C4-C24), a control group (22°C) (C_H_0), and high temperature (37°C) for 4–24 h (H4-H24). In the low-temperature treatment group, the upregulated genes responded slowly to temperature changes; most upregulated genes showed significant upregulation in the 24h low-temperature group, while a few genes, such as *DofPUCHI*, *DofRAP2.11_2*, and *DofERF58*, were already upregulated at 8h of low-temperature treatment. Notably, in the high-temperature treatment group, only a few genes showed upregulation, while high-temperature conditions led to the immediate downregulation of most genes examined, with upregulation occurring only at the later stage (24h) of treatment.

We screened the core genes in the Dof individual to identify those that were differentially expressed(|log_2_FC| ≥ 1, p < 0.05) across the different temperature treatment groups with **Supplementary Figure 2** depicting the expression dynamics of these members. Meanwhile, we established the expression pattern of *DofERF109_2* under different temperature stresses using qRT-PCR, and the results are shown in **Supplementary Figure 3**. The results showed that the expression level of *DofERF109_2* was downregulated under heat stress, whereas no significant difference was observed under cold stress.

### 3.7 Prediction of subcellular localization for the AP2/ERF members

Using the subcellular localization with the highest algorithm score in CELLO v.2.5 as the prediction result, all results are summarized in **Supplementary Table 6**, and the proportions of different localization results are shown in **Figure 1i**. Across the prediction results of all individuals, the nucleus accounted for the largest proportion. Among them, the HXL individual had the highest proportion of members predicted to be in the nucleus (80.26%), while the hs individual had the lowest proportion (62.86%). The second and third most frequent predicted localizations were the chloroplast and cytoplasm, accounting for 14.65% and 7.12%, respectively. These minority members predicted to localize to other subcellular compartments may be a result of functional diversification.

### 3.8 Subcellular localization results of *DofERF109_2*

We observed the results using a laser scanning confocal microscope, as shown in **Figure 5d**. We found that in leaves transformed with the empty vector, fluorescence signals were distributed in both the cytoplasm and the nucleus, whereas in leaves from the experimental group, the fluorescence signal was strictly localized to the nucleus. This result corroborates that *DofERF109_2* is a transcription factor gene.

### 3.9 Experimental results of transient expression of *DofERF109_2* in *D. officinale*

To further investigate the biological function of *DofERF109_2* in cells, we constructed a transient expression plasmid for *DofERF109_2*. The experimental plasmid and the empty vector plasmid were transformed into Agrobacterium tumefaciens and injected into *D. officinale* leaves. Samples were collected on the third day after injection and subjected to qPCR validation. The experimental results, shown in **Figure 5e**, demonstrated that the target gene was significantly upregulated after treatment. Concomitantly, expression of *CHI*, an anthocyanin pathway gene, was downregulated in the experimental group relative to controls. These findings lead us to hypothesize that *DofERF109_2* is involved in modulating anthocyanin synthesis in *D. officinale*.

### 3.10 Experimental results of transient expression of *DofERF109_2* in the tobacco

Based on the *DofMYB47* gene previously screened by our research group, we performed a transient expression assay of *DofERF109_2* in tobacco. On the seventh day after infiltration, the leaves were photographed, and the anthocyanin content in the infiltrated areas was extracted. The results are shown in **Supplementary Figure 4**: yellow spots appeared in the area infiltrated with *DofMYB47*+*AtANS*, whereas the color change was not obvious in the area infiltrated with *DofERF109_2*+*DofMYB47*+*AtANS*. After anthocyanin extraction, the anthocyanin contents of both experimental groups mentioned above were statistically significantly different from that of the positive control, leading us to hypothesize that *DofERF109_2* may inhibit anthocyanin biosynthesis.

## 4 Discussion

### 4.1 Uncovering the AP2/ERFs in *D. officinale*: identification and evolutionary insights

As a large and functionally diverse transcription factor family, AP2/ERF genes are critically involved in plant developmental processes and stress responses. The functional roles of AP2 and RAV subfamily members have been extensively characterized in previous studies (Elliott et al., 1996; Matías-Hernández et al., 2014). Regarding the largest family, ERF, research has found that it can regulate plant organ development. For example, the ERF family member HL6 in rice is involved in regulating signaling pathways for trichome formation, while *SlERF.F5* in tomato can interact with *SlMYC2* in mediating leaf aging and programmed cell death (Sun et al., 2017; Chen et al., 2022). Furthermore, recent findings indicate that members of the ERF subfamily in *A. thaliana* play a bifunctional role in modulating floral organogenesis, influencing both organ quantity and dimensions (Lee et al., 2023). In terms of stress tolerance development, *OsDREB2B* in rice can directly regulate cold-responsive genes, including *COLD1* (Xie et al., 2019; Hu et al., 2024). Meanwhile, ERFs also act as important nodes in the phytohormone regulatory network governing plant stress tolerance, a phenomenon that has been confirmed in both tomato and poplar (Kong et al., 2023; Zhu et al., 2025).

The AP2/ERF transcription factor family has been systematically characterized in numerous angiosperms. Representative member counts include 122 in *A. thaliana*, 139 in rice, 214 in maize, and so on (Nakano et al., 2006; Zhang et al., 2022). In this study, we screened 101, 76, 113, 123, 113, 105, and 113 family members from seven *D. officinale* individuals (Dof, HXL, TM, TP1, TP4, hs, YD). We found that among the 147 AP2/ERF members in *A. thaliana*, 77 members had homologous genes identified in *D. officinale*, while the remaining 70 members did not have detectable homologs. Although nearly half of the *A. thaliana* AP2/ERF members were absent, the members identified in *D. officinale* were still relatively evenly distributed across 14 groups in the phylogenetic tree(with no genes distributed in the ERF-Xb-L group). In summary, compared to *A. thaliana*, the homologous genes in *D. officinale* exhibit a contraction phenomenon. We speculate that this phenomenon may be due to *Orchidaceae* plants retaining a different set of genes compared to Brassicaceae plants during whole genome duplication events; the members without homologous genes found in *A. thaliana* might be those that expanded specifically within the Brassicaceae lineage. Furthermore, we also hypothesize that during rapid gene evolution for adaptation to different environments, specific sequence changes may have occurred, rendering them undetectable by traditional BLASTp and HMMER methods based on *A. thaliana* protein sequences.

Our pangenome-wide analysis revealed that the AP2/ERFs exhibits diverse distribution patterns across distinct *D. officinale* accessions. Based on classical pangenome theory (Loegler et al., 2026), we classified the aforementioned 77 genes into the following categories: a total of 29 core, 28 softcore, 17 dispensable, and 4 private genes were identified. Notably, the core and softcore gene groups exhibited similar abundances, comprising 29 and 28 members. We speculate that the AP2/ERF family maintains relatively high conservation within the *D. officinale* species, while also allowing for gene loss in a few individuals. In the chromosomal localization analysis of the pangenome family, we found that all members in the Dof individual were localized to 18 chromosomes, whereas the members in the other six individuals were distributed across all 19 chromosomes. In pangenome research, overall genetic variation primarily originates from chromosomal structural variations or large-scale presence/absence variations. A study on the *Brassica napus* pangenome found that chromosomal structural variations are tightly linked to ecotype differentiation (Afsharyan et al., 2025), while studies on AP2/ERFs further reveal that the chromosomal distribution of its members is evolutionarily constrained (Deng et al., 2025). Therefore, we hypothesize that due to different habitats, the Dof individual, compared to the others, experienced a chromosome loss event or underwent chromosomal fusion, which compressed genes originally distributed across 19 chromosomes onto 18 chromosomes.

### 4.2 Functional diversity of AP2/ERFs in *D. officinale* pangenome

We observed that several AP2/ERF genes in *D. officinale* exhibit duplication events, resulting in multiple copies per individual. For instance, RAP2.11 from the ERF-V group had 5, 2, 4, 6, 3, 3, and 4 copies in the seven individuals, respectively; similarly, *ERF109*, belonging to the ERF-X group, had 2, 2, 4, 4, 3, 4, and 4 copies, respectively. This phenomenon provides a new perspective for understanding gene evolution. The homologous genes resulting from gene copies often undergo neofunctionalization, subfunctionalization, or pseudogenization (Waterhouse et al., 2011). According to recent findings, genes duplicated through broad-scale events typically maintain overlapping or partitioned functions, whereas duplicates arising from narrow-scale events tend to evolve asymmetrically and acquire novel roles (Almeida-Silva and Van de Peer, 2025). In subsequent analyses, we performed Ka/Ks analysis on homologous genes from different individuals and found that some homologous gene pairs in *D. officinale* had Ka/Ks > 1, such as *ERF36*, *ERF109*, and *CRF5*. These genes may be under positive selection pressure (Roth and Liberles, 2006), i.e., undergoing adaptive evolution, and the aforementioned gene copies might provide the genetic material for this positive selection. Such adaptively evolving genes may be undergoing neofunctionalization. Conversely, most genes with Ka/Ks < 1 are undergoing purifying selection, a phenomenon consistent with findings from studies on other plant gene families: a few genes undergo new functional divergence, while the majority maintain their basic functions (Zhong et al., 2015; Zhang et al., 2024).

Subcellular localization is fundamental for studying gene function. We predicted the subcellular localization of all AP2/ERF members from the seven *D. officinale* individuals and found that 74.87% of the members were localized to the nucleus. The canonical mode of action for AP2/ERFs involves nuclear import and sequence-specific binding to promoter elements such as GCC-box or DRE/CRT, leading to regulation of downstream gene expression (Shao et al., 2025). Consistent with this paradigm, investigation in peanut (*Arachis hypogaea*) revealed that while the majority of members are nuclear-localized, a subset exhibits dual localization in both the nucleus and cytoplasm, revealing potential functional diversification among these variants (Park and Grabau, 2016). In this study, we found that 14.65% of the members were predicted to localize to the chloroplast, and 7.12% to the cytoplasm. Subsequent subcellular localization experiments could be conducted on these minority members to rule out result errors caused by algorithmic limitations.

We documented the expression profiles under temperature stress treatments and observed that these members exhibited both upregulation and downregulation under both conditions. We speculate that this family has undergone functional differentiation in the stress adaptation of *D. officinale*, with different members assuming distinct regulatory roles. Additionally, we found that some members (*DofAIL7*, *DofAIL7_2*, *DofLEP*, *DofERF114*, *DofABI4*) showed no changes in expression levels under either stress condition. These genes may be involved in regulating plant organ development, as such genes typically exhibit stable expression under different conditions (Licausi et al., 2013). Concurrently, we observed that these genes were classified as core genes (*DofAIL7*, *DofAIL7_2*, *DofLEP*) and softcore genes (*DofERF114*, *DofABI4*). This indicates a conserved distribution of these genes across different individuals, a phenomenon that further supports the notion that these members may function as housekeeping genes.

### 4.3 *DofERF109_2* regulates anthocyanin biosynthesis in *D. officinale*

Anthocyanins, a type of flavonoid compound, possess antioxidant functions and inhibit pathogen growth in plants. Additionally, they act as plant pigments, aiding entomophilous plants in attracting insects (Shen et al., 2022; He et al., 2023). Evidence from apple indicates that the AP2/ERFs contribute to anthocyanin production through the regulatory influence of various individual members, including *MdERF1B* (Zhang et al., 2018), *MdERF3* (An et al., 2018), *MdERF38* (An et al., 2020), and *MdERF109* (Ma et al., 2021).

We found that *ERF109*, an AP2/ERF member in *D. officinale*, belongs to the core genes and exhibits a Ka/Ks > 1 phenomenon across different individuals. In the transposable element analysis, transposon fragments were detected in the HXL and hs individuals but were absent in the others. We hypothesize that the insertion of this transposon might be a result of recent evolution and has not yet become fixed within the *D. officinale* population. Subsequently, we observed that it was differentially expressed in the high-temperature and control transcriptomes and showed upregulation in the 24-hour low-temperature treatment group, leading us to speculate that it may be involved in plant stress responses. Furthermore, we screened *DofERF109_2* in *D. officinale*, which is homologous to *MdERF109*, a member involved in regulating anthocyanin biosynthesis in apple. Therefore, we conducted subcellular localization and subsequent functional validation experiments on *DofERF109_2*.

In this study, nuclear localization of *DofERF109_2* was demonstrated through subcellular imaging analysis. In subsequent functional validation, we analyzed the expression pattern of *DofERF109_2* under cold and heat stress and found that its expression level was downregulated under heat stress (30 °C). Furthermore, we found that the addition of *DofERF109_2* inhibits anthocyanin biosynthesis in tobacco leaves, and simultaneously, transient expression of *DofERF109_2* in *D. officinale* suppresses the relative expression level of *DofCHI*. Other studies have shown that knockout or suppression of *CHI* results in elevated chalcone levels decreased flavonoid synthesis (Zhao et al., 2021). We hypothesize that *DofERF109_2* may negatively regulate anthocyanin synthesis, a negative regulatory role for this gene family that has also been reported in red-skinned pears (Sun et al., 2023). In future studies, we will employ assays to validate the interacting proteins and target genes of *DofERF109_2* within the anthocyanin biosynthesis pathway. These investigations will contribute to a more comprehensive understanding of the regulatory network in the anthocyanin biosynthesis pathway of *D. officinale*.

## 5 Conclusions

By analyzing seven *D. officinale* genomes at the pangenome level, we identified 633 genes belonging to the AP2/ERFs. These members were classified into 14 groups within the phylogenetic tree, and 74.84% of them were predicted to localize to the nucleus. At the pangenome family level, Our classification scheme, which considered gene presence across individuals, revealed core (n=29), softcore (n=28), dispensable (n=17), and private (n=3) genes. Among the core, softcore, and dispensable genes, all categories contained members with transposable element fragments. With the exception of the Dof individual, the AP2/ERF members in all other individuals (HXL, TM, TP1, TP4, hs, YD) were located on the 19 chromosomes. In the selection pressure analysis conducted on homologous genes (excluding private genes), 25 genes under positive selection were identified. Regarding gene expression under different temperatures, we screened and obtained 8 differentially expressed members in the low-temperature group and 12 members in the high-temperature group. From the above integrative analysis, *DofERF109_2* emerged as a candidate for further investigation. Experimental evidence revealed its nuclear localization, and its transient expression inhibited anthocyanin biosynthesis in tobacco leaves while downregulating the structural gene *DofCHI* involved in anthocyanin biosynthesis in *D. officinale*.

## Data Availability

The datasets presented in this study can be found in online repositories. The names of the repository/repositories and accession number(s) can be found in the article/**Supplementary Material**.
