# Supplementary figures and images for: Identification and analysis of the AP2/ERF gene family in Dendrobium officinale based on pan-genome and functional characterization of DofERF109_2
Source: Front Plant Sci. 2026 Jun 10;17:1834268. doi: 10.3389/fpls.2026.1834268 (PMC13291111; doi:10.3389/fpls.2026.1834268)

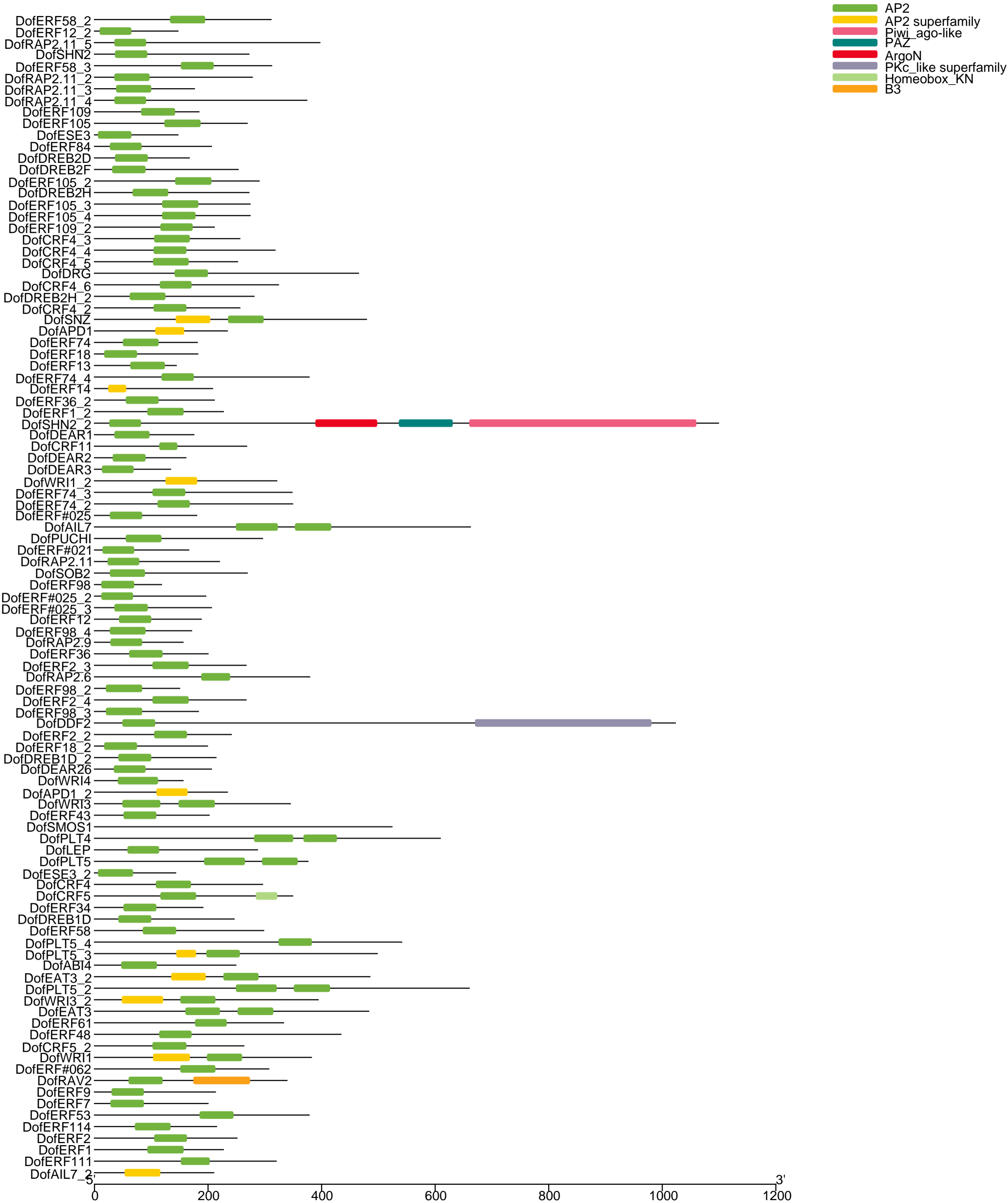

Supplement: Supplementary file 1 [file DataSheet1.zip › CDD Figure/Dof CDD.pdf]

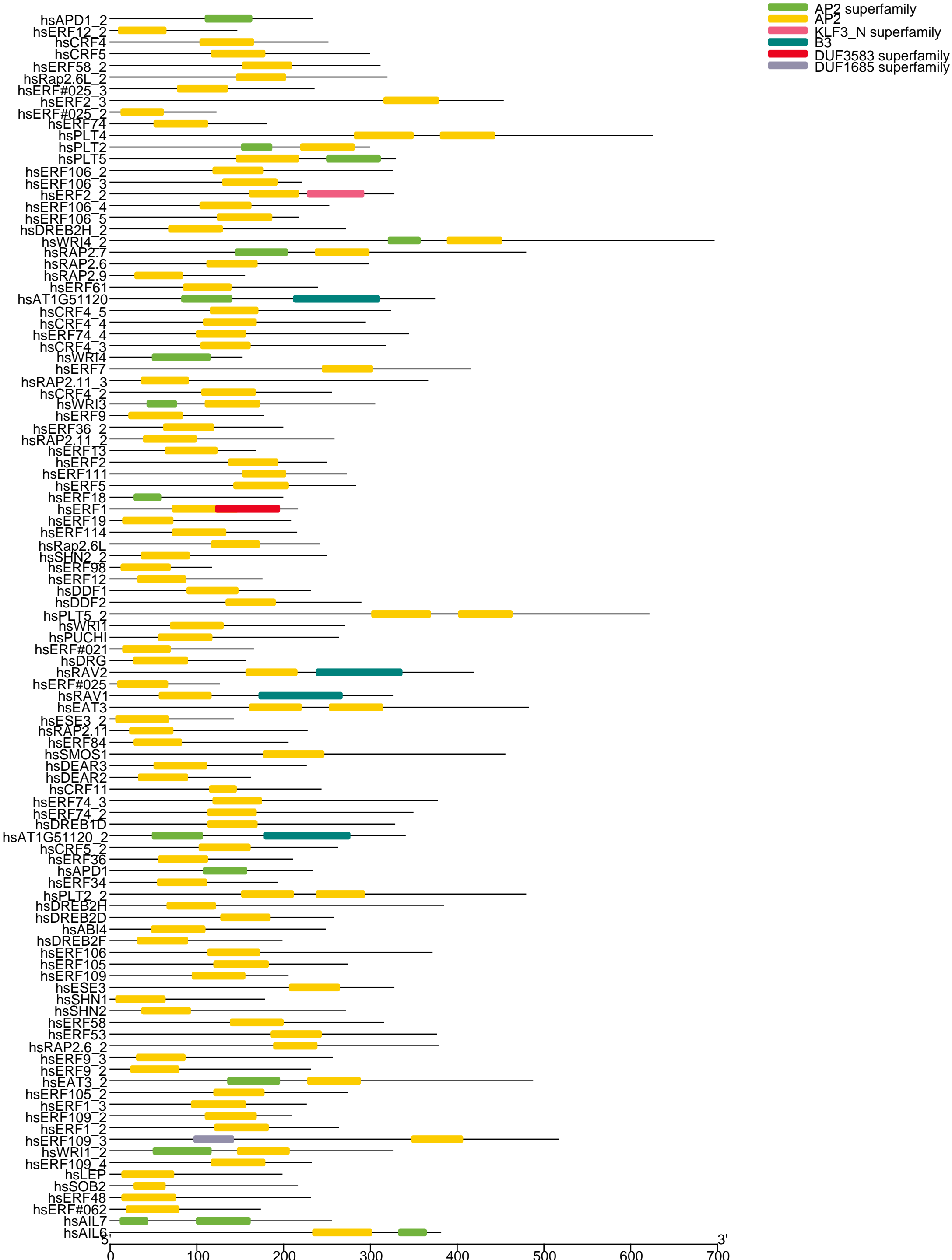

Supplement: Supplementary file 1 [file DataSheet1.zip › CDD Figure/hs CDD.pdf]

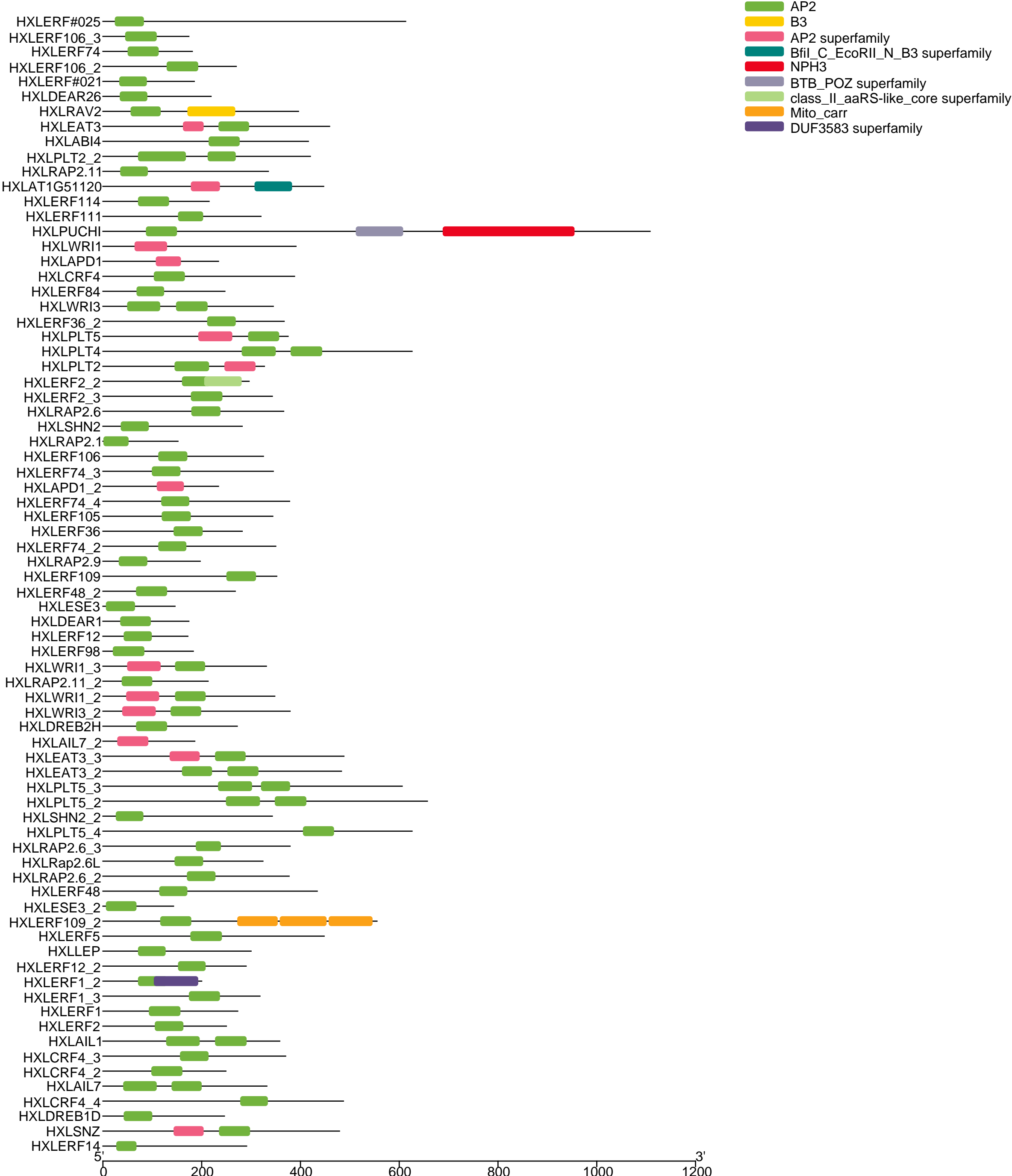

Supplement: Supplementary file 1 [file DataSheet1.zip › CDD Figure/HXL CDD.pdf]

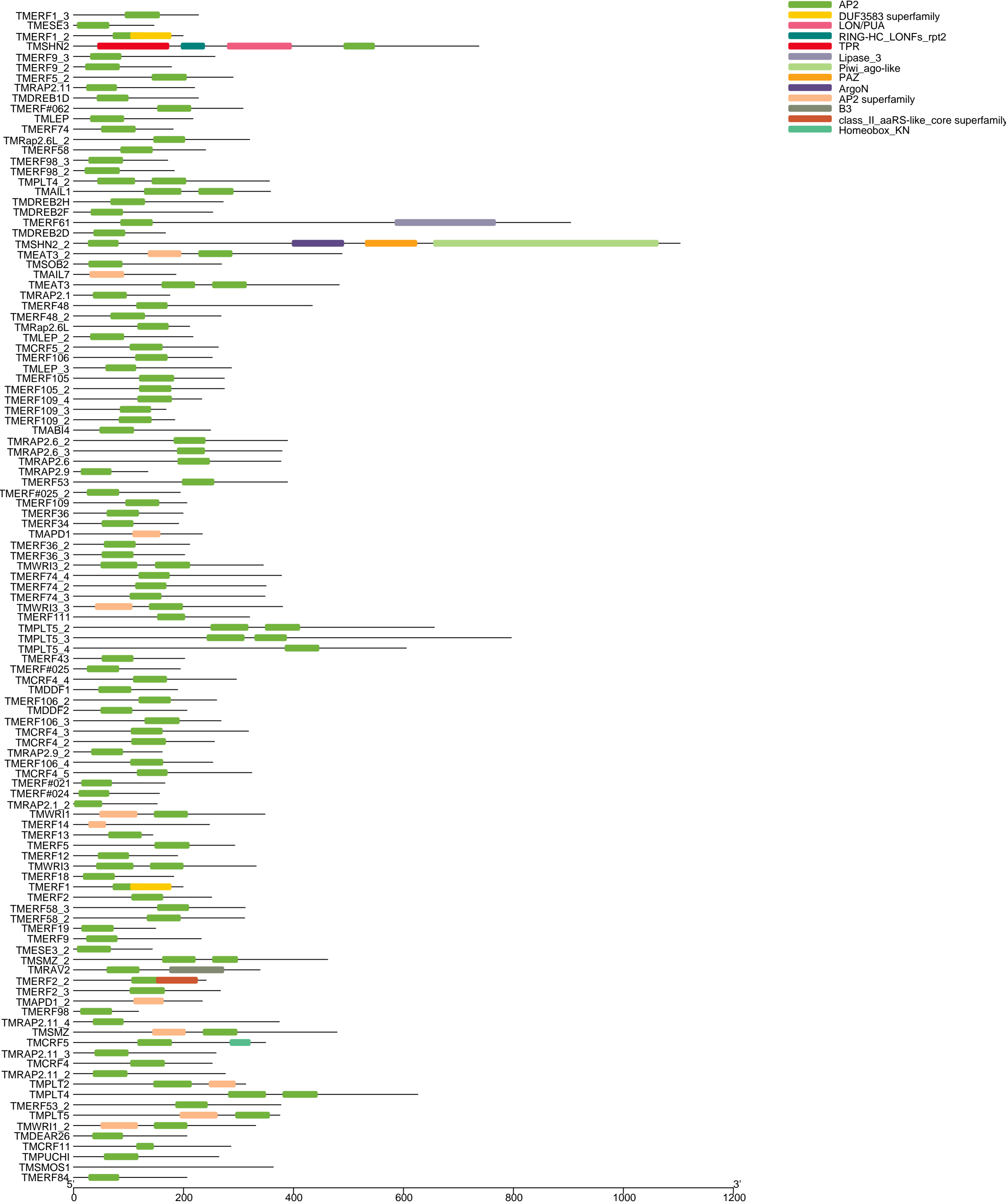

Supplement: Supplementary file 1 [file DataSheet1.zip › CDD Figure/TM CDD.pdf]

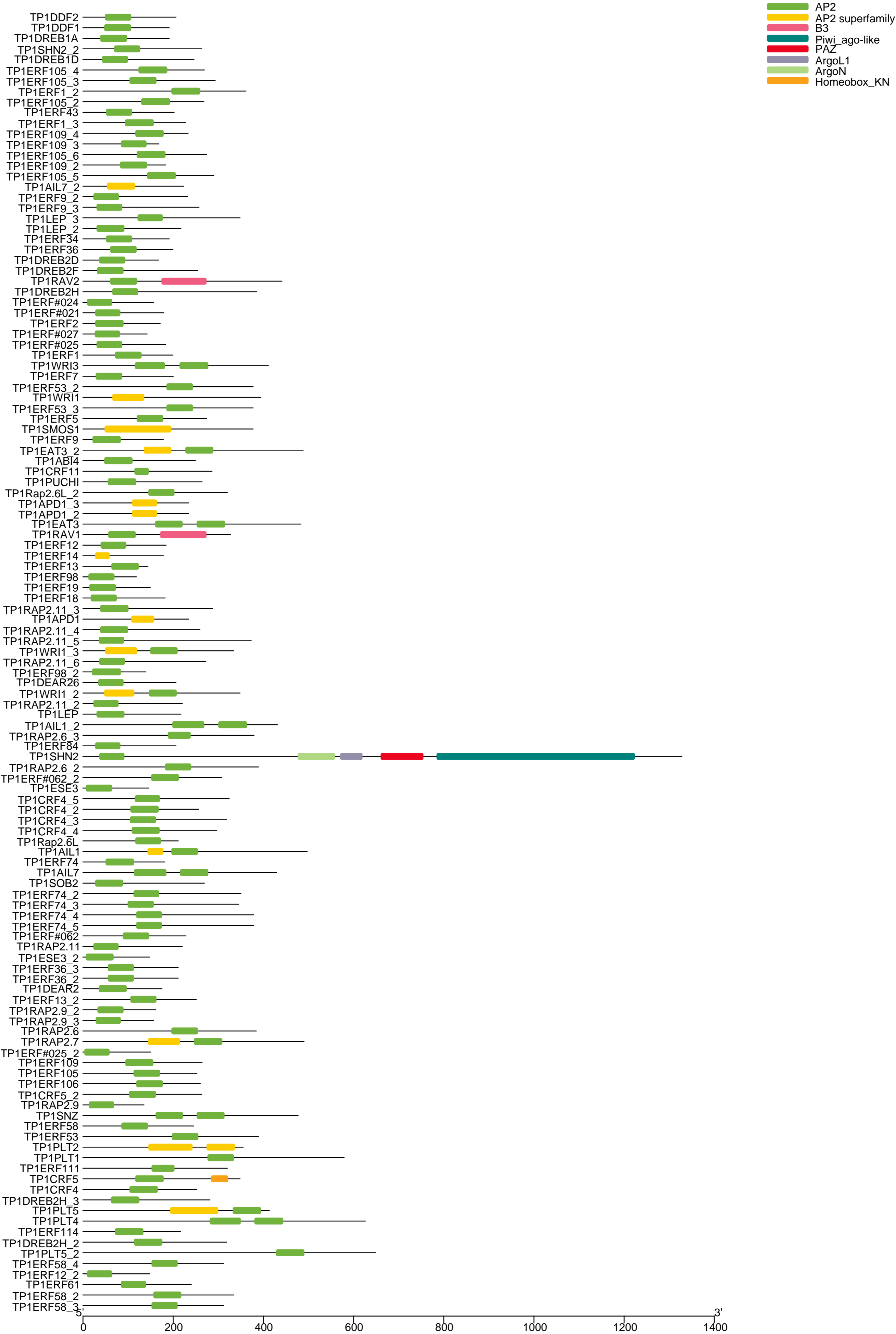

Supplement: Supplementary file 1 [file DataSheet1.zip › CDD Figure/TP1 CDD.pdf]

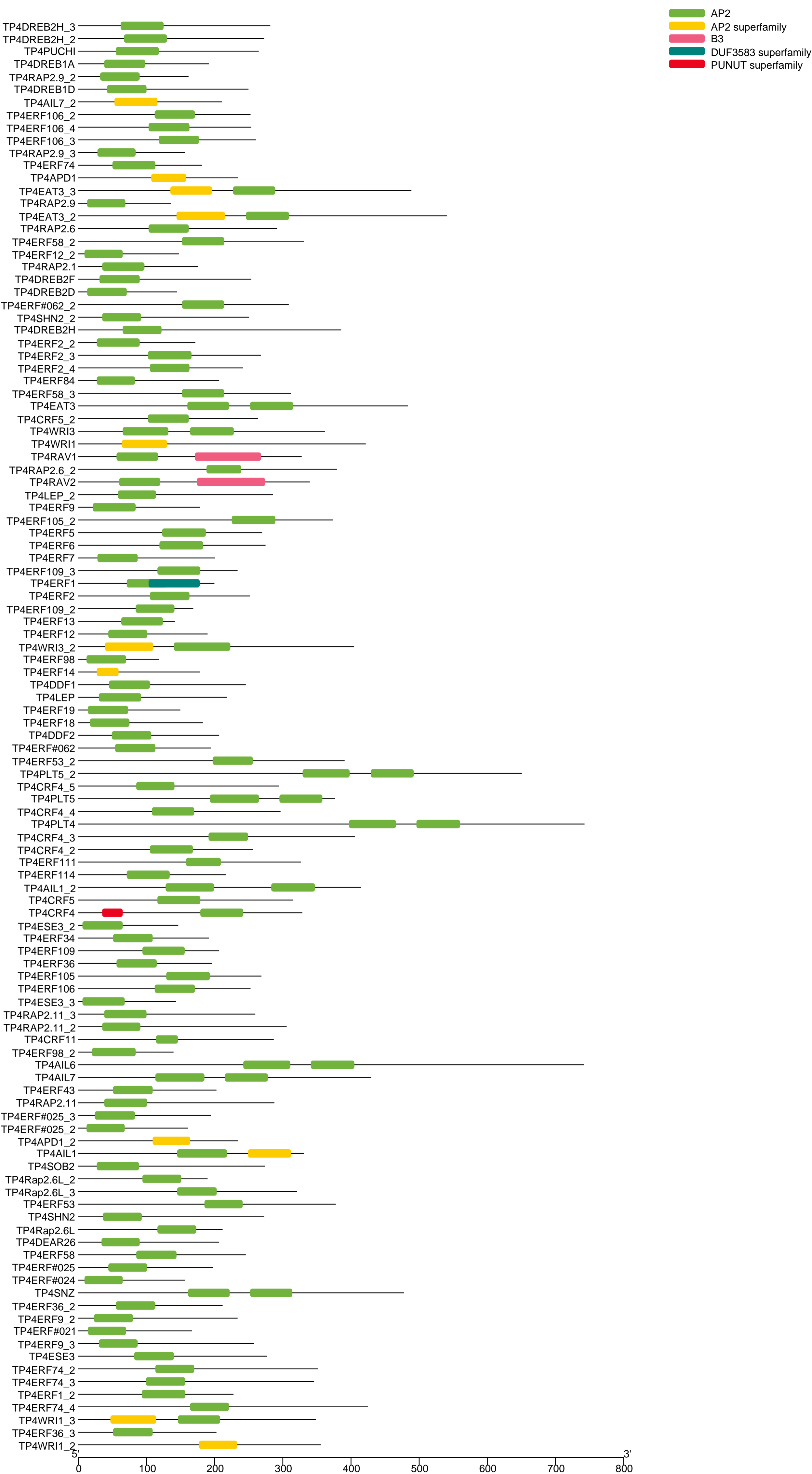

Supplement: Supplementary file 1 [file DataSheet1.zip › CDD Figure/TP4 CDD.pdf]

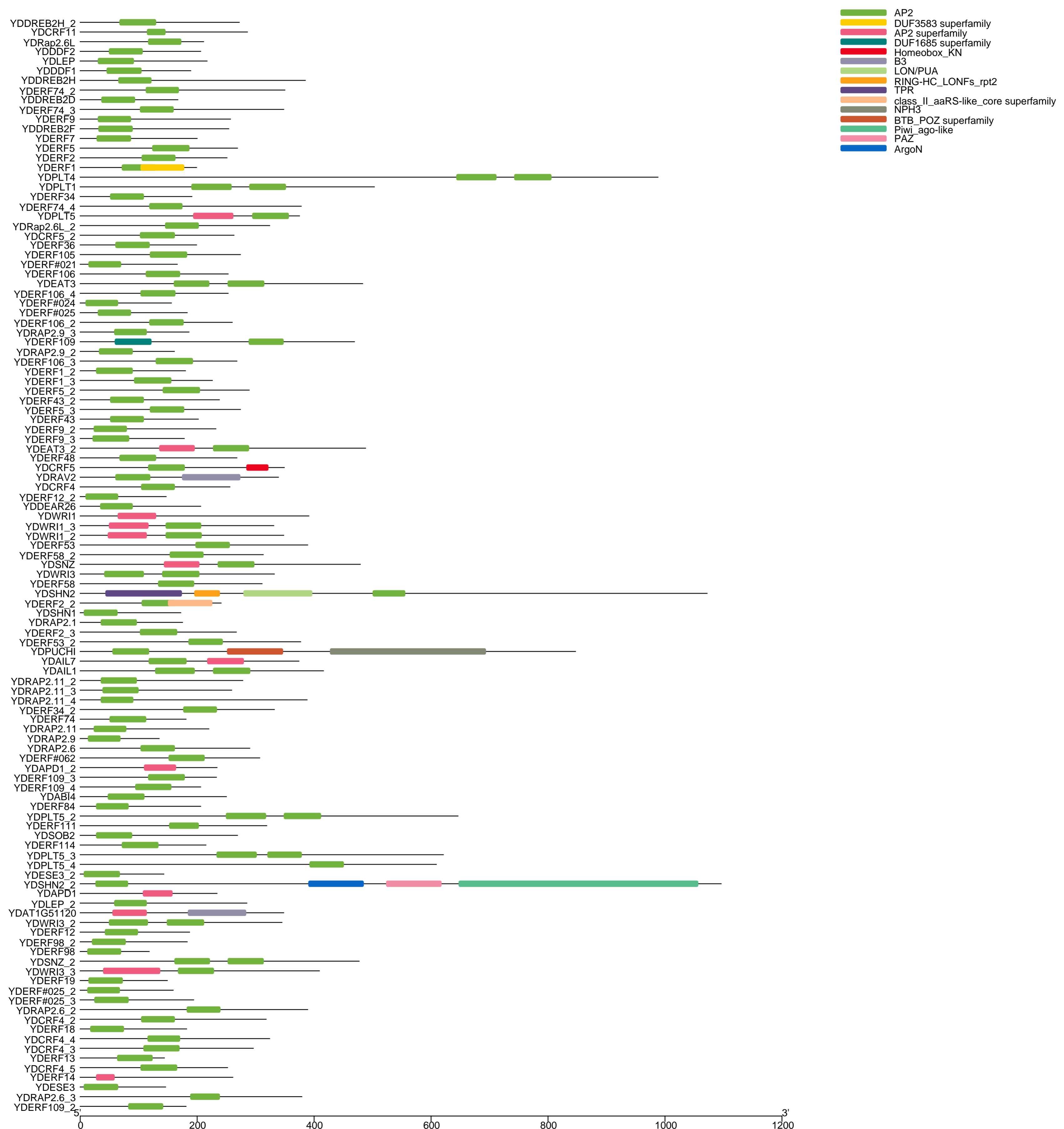

Supplement: Supplementary file 1 [file DataSheet1.zip › CDD Figure/YD CDD.pdf]

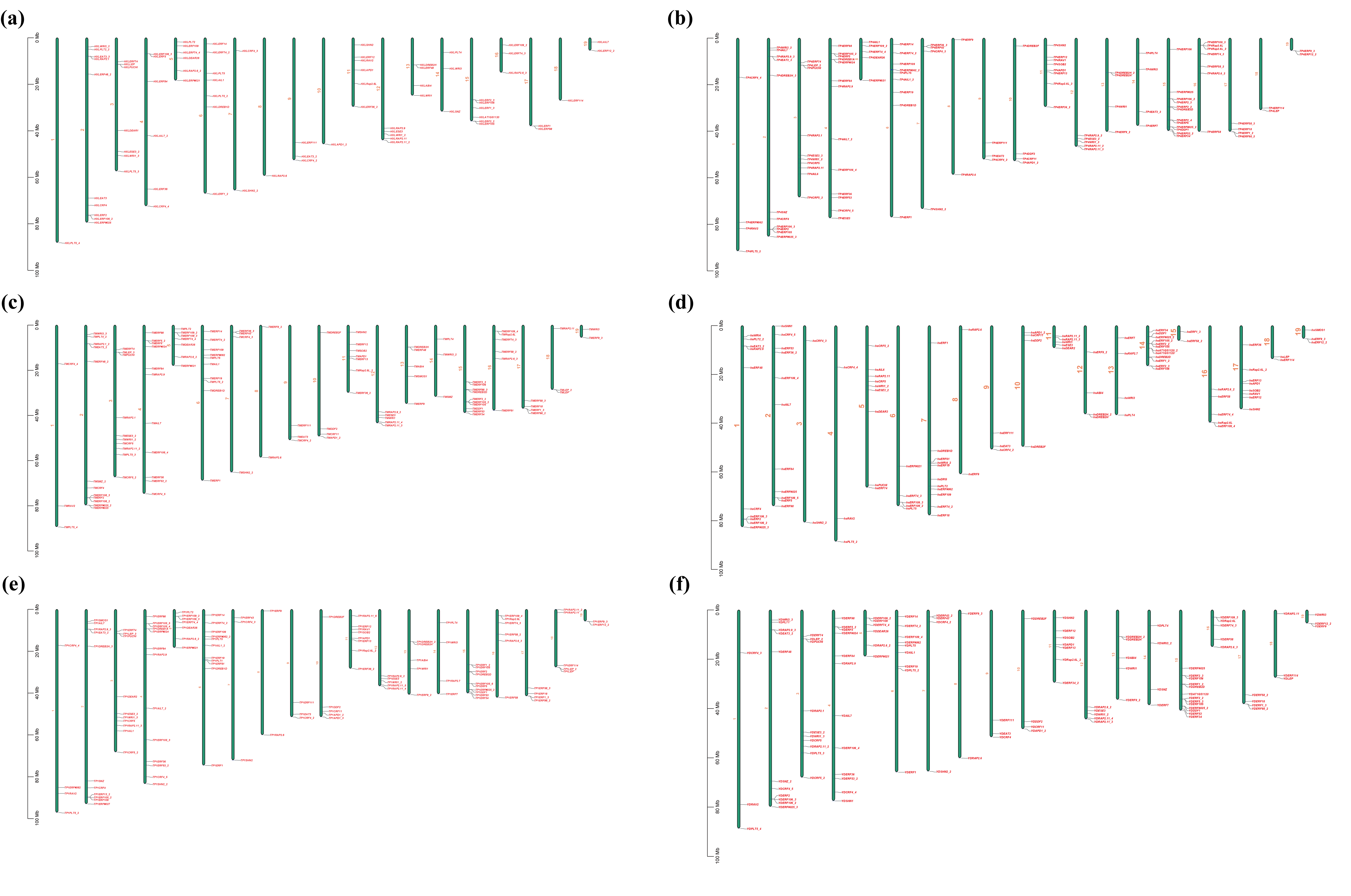

Supplement: Supplementary file 2 [file Image1.tif]

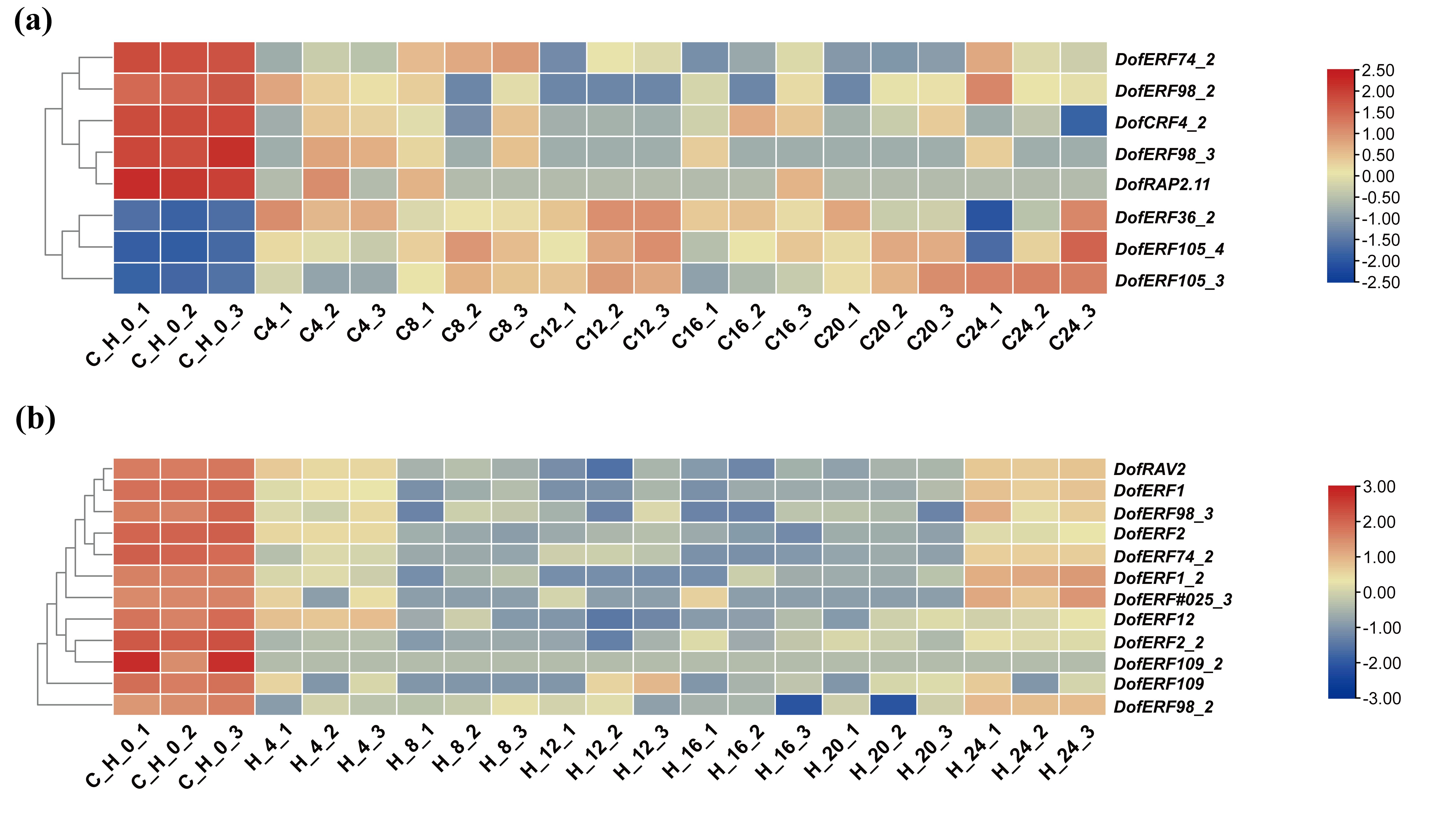

Supplement: Supplementary file 3 [file Image2.tif]

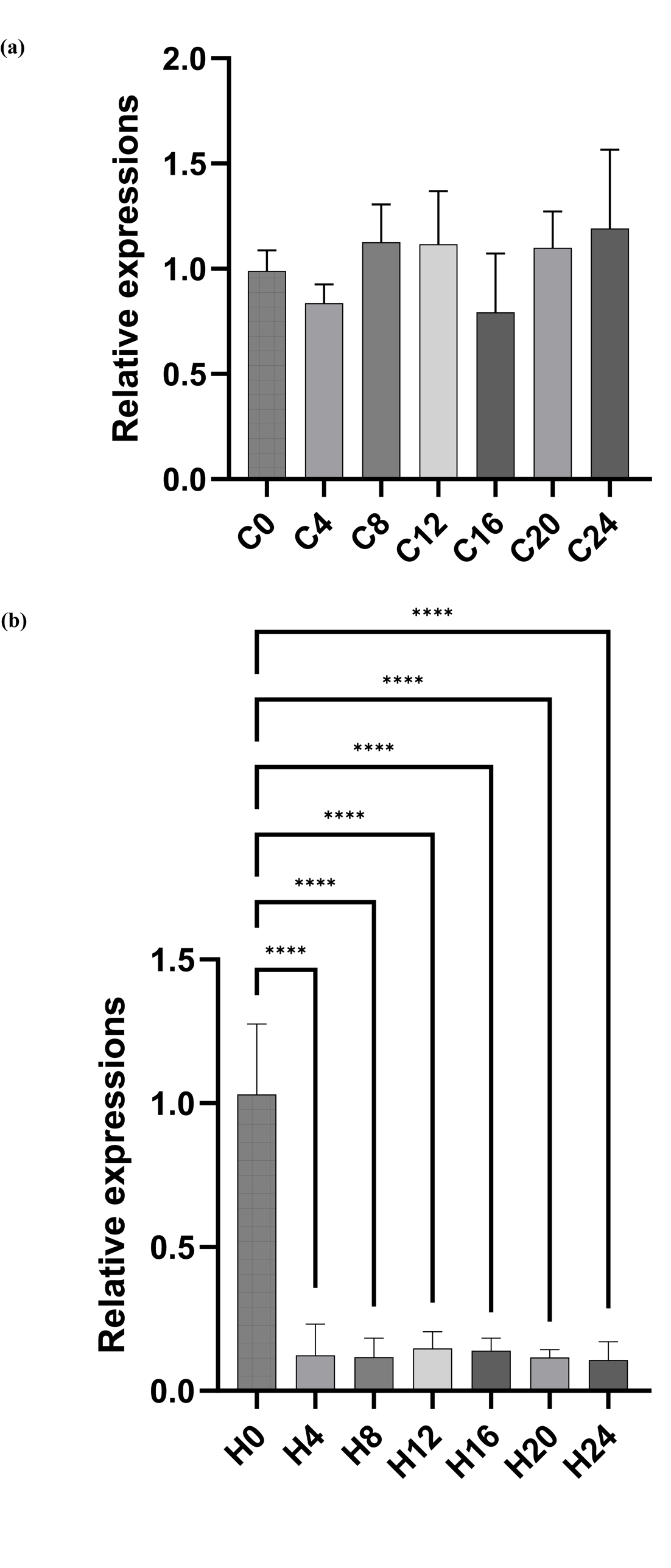

Supplement: Supplementary file 4 [file Image3.tif]
